# Impact of fortified biscuits on micronutrient deficiencies among primary school children in Bangladesh

**DOI:** 10.1371/journal.pone.0174673

**Published:** 2017-04-05

**Authors:** Alayne M. Adams, Rushdia Ahmed, A. H. M. Mahbub Latif, Sabrina Rasheed, Sumon K. Das, Enamul Hasib, Fahmida Dil Farzana, Farzana Ferdous, Shahnawaz Ahmed, ASG Faruque

**Affiliations:** 1 Health Systems and Population Studies Division, International Centre for Diarrhoeal Disease Research, Bangladesh (icddr,b), Dhaka, Bangladesh; 2 James P. Grant School of Public Health, BRAC University, Dhaka, Bangladesh; 3 Department of International Health, Georgetown University, Washington DC, United States of America; 4 Institute of Statistical Research and Training, University of Dhaka, Bangladesh; 5 Centre for Clinical Epidemiology, St. Luke’s International University, Tokyo, Japan; 6 Nutrition and Clinical Services Division, International Centre for Diarrhoeal Disease Research, Bangladesh (icddr,b), Dhaka, Bangladesh; 7 School of Public Health, The University of Queensland, Brisbane, Queensland, Australia; 8 Graduate School of Comprehensive Human Sciences, University of Tsukuba, Tsukuba, Ibaraki, Japan; Universidade de Sao Paulo, BRAZIL

## Abstract

**Background:**

Micronutrient deficiencies can compromise the development potential of school-aged children, and their later health and productivity as adults. School feeding and school-based fortification approaches have been utilized globally to redress nutritional deficiencies in this age group.

**Objective:**

We explored the acceptability and micronutrient impact of a Bangladesh Government supported school-based micronutrient fortification program for children attending rural primary schools in 10 disadvantaged sub-districts.

**Methods:**

We applied a mixed methods approach. The quantitative component assessed the impact of micronutrient fortification on 351 children aged 6–11 years using a cohort pre-post research design with a control group. The qualitative component explored the acceptability of the intervention using focus group discussions, body mapping and semi-structured interviews with teachers, school-going children and school authorities.

**Results:**

Daily consumption of fortified biscuits by primary school children had a significant positive impact on mean levels of iron, folic acid, vitamin B12, retinol and vitamin D controlling for sex, baseline deficiency status, CRP, and H. pylori. Levels of anemia and vitamin D deficiency were also significantly reduced. Qualitative findings indicated the widespread acceptability of the daily biscuit. Teachers perceived students to be more attentive in class, less tired, and some attributed better school performance to biscuit consumption. Children reported similar improvements in concentration and energy levels.

**Conclusions:**

This study is among the first in Bangladesh to comprehensively assess a school-based fortification program in terms of its acceptability and impact on micronutrient status of children aged 6–11 years of age. While results strongly support this modality of school feeding, research on the cognitive impacts of micronutrient fortified biscuits will help clarify the case for scaled-up investments in school- based feeding program in Bangladesh and other low and middle income countries.

## Introduction

It is well established that malnutrition in early life can compromise the development potential of children, and their later health and productivity as adults [1]. In Bangladesh, extraordinary gains in child survival have occurred since independence in 1971, with progressive yet slightly more modest improvements in rates of malnutrition over the same period. Recent policy and programs focus on the first 1000 days, however, have yielded further gains in the nutritional status of under-fives through intensive efforts around breastfeeding, complementary feeding and micronutrient supplementation [2]. Comparatively less attention has focused on nutritional deficiency among young school aged children, an age group that is also characterized by rapid growth and development [3].

Nationally representative data on children aged 6–14 in Bangladesh have identified deficiencies in Vitamin A (20.9%), Iodine (40%), and Iron (3.9%), and rates of Anemia and Iron Deficiency Anemia in the order of 19.1% and 1.3% respectively [4]. Lack of diversity of diet is a major determinant of children’s poor micronutrient profile, with deficiencies linked to increased risk of infection, stunted growth and diminished cognitive performance [2, S1 Appendix]. For these reasons, interventions to address micronutrient deficiencies in school-aged children have been proposed as a means of improving their health, growth and cognitive performance.

### School feeding interventions

To address the nutritional needs of young school going children and provide social protection to families, global efforts have largely focused on school feeding and school-based fortification approaches [5]. There are several modalities of school feeding, which can be classified into two main groups: take home rations, and in-school feeding programs involving the provision of meals or snacks such as biscuits. In addition to their contribution to social protection, there is substantial evidence that if effectively implemented, school feeding programs can increase school attendance, cognition, attention span, and educational achievement [6]. Evidence is even more persuasive when accompanied by complementary actions such as de-worming and micronutrient fortification or supplementation [5].

A number of studies have explored the impact of fortification among primary school children in South Africa and Vietnam. Biscuits fortified with multiple micronutrients were found to have a significant treatment effect for serum retinol, serum iron, urinary iodine, serum ferritin, and hemoglobin [7, 8]. Significant impacts on anemia prevalence [8, 9] and deficiencies of zinc and iodine were also noted [8]. In Bangladesh, a randomized control trial of a primary school-based fortification program in 2002 showed a 4.3% or 0.62 unit of Body Mass Index (BMI) increase in the treatment group compared to the control group, and a 15.7% improvement in Grade 4 test scores [10]. Our study contributes to this literature by investigating the acceptability and micronutrient impact of a similar school-based fortification program in Bangladesh implemented in selected poor and disadvantaged sub-districts throughout the country.

## Methods

### The intervention

The Bangladesh government, with support from the European Union, initiated a pilot school feeding program in 10 disadvantaged sub-districts which consisted of the daily administration of a packet of fortified biscuit to all primary school-going children aged 6–11 years. During the roll-out of the pilot intervention, our research team measured the micronutrient status of children aged 6–11 who attended primary schools in the program area. One year later we undertook repeat measures on the same children. Similar measurements were made on a control group of primary school children living in adjacent sub-districts where the program had not been implemented.

Biscuit ingredients were: wheat flour (69% by weight); sugar (12%); vegetable fat (hydrogenated-75% & liquid-25%- 13%); soya flour (6%); iodized salt (0.5%); leavening agent (1.0%) and micronutrient premix (1.5 kg premix in 998.5 kg biscuit dough). The fortified biscuit was prepared to provide 300 kcal per single 75 gm packet (approximately 15% of daily calorie requirements), and a range of micronutrients contributing to about 75% of the daily requirements of vitamin A, folate, iron, iodine, zinc and magnesium (Table 1).

**Table 1:** Nutrient composition of fortified biscuits per 100 gm.

Nutrients/Micronutrients	Amount
Energy, kcal	450
Moisture (maximum)	4.5%
Protein, g	10–15
Fat, g	15
Calcium, mg	212.5–287.5
Magnesium, mg	127.5–172.5
Vitamin A (retinol), mcg	212.5–287.5
Vitamin D, mcg	1.615–2.185
Vitamin E, mg	4.25–5.75
Vitamin B1, mg	0.425–0.575
Vitamin B2, mg	0.595–0.805
Vitamin B3 (niacin), mg	5.1–6.9
Vitamin B5 (pantothenic acid), mg	2.55–3.45
Vitamin B6, mg	0.85–1.15
Vitamin B12, mcg	0.425–0.575
Folic Acid, mcg	680–920
Vitamin C, mg	17.0–23.0
Iron, mg	9.35–12.65
Iodine, mcg	63.75–86.25
Zinc, mg	7.00–8.00

Biscuit production occurred in five factories close to pilot project areas. Production costs were approximately 6.5 taka (less than US 9 cents) per 75g packet. Biscuits were shipped to NGO warehouses and distributed to primary schools. A regular supply of biscuits was maintained with the exception of a brief period of political unrest, and seasonal flooding in certain sub-districts. Each carton contained 100 packets of biscuits, and a log/register in every school was maintained to keep count of cartons received and distributed. Class teachers were responsible for distributing biscuits among their students. According to teachers, there were few reports of damage associated with transportation, storage, or pests, and consumption of the biscuit was enthusiastic and virtually universal among students.

### Study design

This was a mixed methods study. The quantitative component consisted of a cohort pre-post research design with a control group. One year into implementation, intervention impact was assessed by comparing baseline and endline measures in the same population of children. Quantitative data collection was undertaken by means of a structured survey questionnaire to collect data at individual (child), household (mother) and institutional (school) levels. Micronutrient measurements were conducted on a subsample of these children. The qualitative component explored the acceptability of the intervention by means of focus group discussions (FGD) with teachers; FGD and body mapping with children, and semi-structured interviews with school authorities. This paper focuses on results from the subsample of children from whom blood samples were collected.

### The quantitative study

#### Sampling strategy

A cluster randomized approach to sampling was employed in which schools represented clusters. Among the 10 sub-districts where the pilot fortification program was initiated, 7 sub-districts were randomly chosen, and from each of these, one school was randomly selected into the study. The control group consisted of 7 schools selected randomly from 7 adjacent sub-districts where there was no intervention, but which shared the basic socio-economic background characteristics of intervention schools. A multi-stage sampling design was followed to randomly select children. To enroll a sufficient number of school children, lists of students in grades 1–3 were obtained from the school authority. Children were identified randomly from these lists through computer-generated random numbers ensuring that an equal number of boys and girls were selected. Children whose cognitive abilities were challenged by autism, mental issues or iodine deficiency were excluded from the study. The same children were interviewed at baseline and endline.

#### Data collection

The baseline survey took place between September 12–26, 2011, and end-line measurements occurred 14 months later, from November 5–17, 2012. Initial biological samples were collected two months following the baseline. The qualitative component was conducted ten months into the intervention (late June) in the same year as the endline.

#### Sample size calculation

To detect pre-post differences in micronutrient levels in children receiving the intervention, as well as differences between intervention and control groups, we calculated sample size using the method for comparing two groups across time [11]. A minimum sample size for each micronutrient indicator was initially identified. Among these, Hemoglobin (Hb) require the largest sample size: to detect a difference of 0.17 units at a 5% level of significance with 80% power, and a population standard deviation of 1 and correlation coefficient of 0.5, the minimum number of students needed for each group was 161. At baseline, a sample of 191 and 177 students were recruited into intervention and control groups respectively to account for probable loss to follow-up.

### The survey

Survey data were collected by means of questionnaires from students, mothers, and teachers. The student questionnaire was comprised of a series of questions that assessed what they have eaten recently, as well perceptions of satiety, hunger, energy levels, ability to concentrate, and whether there had been recent school absences.

The mother’s questionnaire covered background information concerning household food security and socio-economic status, as well as information on the child’s eating practices, recent morbidity, and perceived energy level, concentration and irritability.

The teacher’s questionnaire sought information about their perceptions and experience regarding the behavior of children in the classroom.

#### Anthropometry

Measures of student height and weight were taken by a team of trained and experienced field workers. Weight was measured to the nearest 100 g using a digital scale. Height measured within 0.1 cm using a locally constructed instrument in which a plastic tape measure was extended between a footplate and head bar. The mean of three consecutive measurements of height and weight was considered as the observed value at the time of data analysis [12].

#### Biochemical measures

For each student enrolled in the study, a 2 ml blood sample was obtained by venipuncture using aseptic technique, and biochemical analyses performed to identify anemia and other micronutrient deficiencies. Urine samples were also collected to assess urinary iodine. Blood and urine samples were collected on the school premises, and immediately placed in an aluminum foil-covered vial to minimize light exposure and kept in a foam rack at room temperature until clotting occurred. All samples were centrifuged in a makeshift laboratory. Within 8–12 hours of collection, the separated serum samples were transported for storage and analysis in tightly-packed Eppendorf tubes maintaining a cold-chain that ranged between temperature 4 and 8°C in cool boxes with ice-packs to the Nutritional Biochemistry Laboratory of icddr,b and stored at -20°C until analysis. Fast-moving motor vehicles were used for quick transportation of the specimens, and when necessary, timely initial processing occurred at a makeshift laboratory in the field. Biochemical analyses were performed by highly skilled laboratory technicians at icddr,b using state of the art equipment.

#### Definitions

The calculations and cutoffs employed to determine deficiency levels related to anemia, iron deficiency anemia, plasma ferritin, folic acid, iodine, vitamins A, B12 and D, and zinc are presented in Table 2. A plasma concentration of CRP 0.10 mg/L or >10 mg/L was used to indicate an acute phase response, and optical density levels of H. pylori equal to or greater than 0.140 were considered positive for H. pylori infection.

**Table 2:** Micronutrient deficiency cut-offs for children.

Deficiency	Age group	Reference
Anemia	5–11 years	Hemoglobin <11.50 g/dL [13]
Iron deficiency anemia (IDA)	Children < 5 years	Hemoglobin <11.50 g/dL; Ferritin <15.00 ng/mL [14]
Folic acid deficiency	Children 9–11 years	Serum Folate < 3.0 ng/mL [15]
Urinary iodine deficiency	Children ≥ 6 years	Urine Iodine < 100.00 μg/L [16]
Vitamin A deficiency	Children < 5 years	Serum Retinol ≤ 0.70 μmol/L [17]
Vitamin B12 deficiency	Children 9–11 years	Serum Vitamin B12 < 200 pg/mL [15]
Vitamin D deficiency	Children 9–12 years	Serum Vitamin D < 37.00 nmol/L [18]
Zinc deficiency	Children < 10 years	Serum Zinc < 0.65 mg/L [19]

#### Statistical analysis

In this study, the effect of fortified biscuits (treatment) was assessed for different micronutrients separately after adjusting for important covariates, namely mother’s education, household wealth index, student’s gender and age, presence or absence of H. pylori and C-reactive Protein, and consumption of different food types. We used the Generalized Estimating Equation (GEE) [20] method to estimate the treatment effect after adjusting for important covariates because micronutrient measurements obtained from each student are assumed to be correlated. Micronutrients are measured in continuous scale and mean log-micronutrients are modeled with an identity link function. Separate log-binomial regression models [21] are used to estimate the prevalence of different micronutrient deficiencies at endline after adjusting for important baseline covariates, namely the baseline status of corresponding deficiencies, gender, presence or absence of H. pylori at baseline, and levels of C-reactive protein. Large sample theory of statistical inference is used to test the significance of the regression parameters in this analysis.

### The qualitative study

Using a purposive sampling approach, in each school 2 FGDs were conducted: one involving teachers and one the other consisting of 8 to 10 students from Classes 2 to 4. Each session lasted 40–45 minutes, and discussion was digitally recorded with concurrent note taking. In addition to group discussions, body mapping was conducted to help students describe the perceived physical and mental effects of the biscuit on the body in a systematic and participatory manner. An outline of a student volunteer’s body was traced on white paper by another child, and taped to the wall. Children were then asked to think about how their bodies feel when they eat biscuits, and then indicate where on the body map these effects occur. Positive effects were shown using a green marker and negative effects with a red marker. Semi-structured interviews with school authorities and headmasters were also conducted using checklists and questionnaires.

Audio recordings of focus group and interview data were transcribed verbatim, and analyzed using ATLAS.ti. A priori codes were applied that linked to specific research questions, and code-specific reports were produced to facilitate systematic analysis. Before starting each session, oral consent was obtained regarding agreement to participate and willingness to have the session to be audio recorded.

### Conceptual framework

For the quantitative analysis we explored the impact of a primary school feeding program on children’s micronutrient status. We used the UNICEF conceptual framework for malnutrition to select variables to include in our regression model [22]. The dependent variable was the micronutrient status for various micronutrients. In terms of immediate causes we considered child factors such as infection (C-reactive protein and H. pylori) and dietary intake (7 day recall for food groups) [23]. In terms of underlying causes we included both child-level factors (age, sex, height, weight, BMI) and household-level factors (parental education and wealth index). The independent variables were collected via the mother’s questionnaire.

### Ethical clearance

The project was approved by the Research Review Committee and Ethical Review Committee of International Centre for Diarrhoeal Disease Research, Bangladesh (icddr,b). Written consent was obtained from school authorities, guardians and their children for both data and biological specimen collection. For blood collection, a team of experienced technicians was deployed to ensure that the blood collection procedures were safe and accurate.

## Results

### Quantitative results

A total of 368 primary school children and their families were enrolled at baseline. At endline, sample size dropped to 351 students (intervention 189, control 162) due to school absences during the day of data collection, children having moved out of the area since the baseline, and other unknown reasons. As described by the conceptual model, our analysis sought to assess the impact of biscuit consumption on student’s micronutrient status, controlling for a variety of student and household-level covariates. As shown in Table 3, at baseline, the characteristics of primary school students in intervention and control groups were largely similar. At the household level, however, mothers were better educated in the control group whereas fathers were better educated in the intervention group (Table 3).

**Table 3:** Baseline characteristics of primary school children in intervention and control groups*.

Characteristics		Intervention	Control
		(191)	(177)
		Mean ± sd	Mean ± sd
***Immediate causes***			
C-reactive protein(mg/L)		0.50 ± 0.78	1.18 ± 4.37
H. pylori		70.2%	54.2%
Food consumed (days/last 7 days)	Rice	6.93 ± 0.51	7.00 ± 0.00
	Fish	3.76 ± 2.35	3.45 ± 2.45
	Dal	1.93 ± 1.91	2.29 ± 2.13
	Meat	0.80 ± 1.38	0.51 ± 1.06
	Egg	1.22 ± 1.74	1.04 ± 1.54
	DGLV	2.25 ± 2.04	1.83 ± 1.61
	YOFV	1.23 ± 1.69	1.14 ± 2.06
	Milk	1.47 ± 2.60	1.18 ± 2.46
***Underlying causes***			
Age (years)		9.08 ± 1.28	8.74 ± 1.31
Sex	Male	45.5%	47.5%
Anthropometry	Height (cm)	125.90 ± 7.78	123.19 ± 7.73
	Weight (kg)	22.69 ± 4.06	21.47 ± 3.94
	BMI (wt/ht^2^)	14.20 ± 1.25	14.04 ± 1.26
Wealth index	Lowest	19.9%	23.7%
	Second	25.1%	20.9%
	Middle	16.2%	20.3%
	Fourth	15.2%	19.8%
	Highest	23.6%	15.3%
Mother’s schooling	Years	2.87 ± 3.37	3.49 ± 3.30
Father’s schooling	Years	3.64 ± 4.12	3.12 ± 3.84

*Values are mean± sd or %

DGLV = dark green leafy vegetables, YOFV = yellow orange fruits and vegetables

When the micronutrient status of primary school children at baseline and endline were compared, we observed a secular increase in average micronutrient levels over time in both intervention and control groups (Table 4), with exception of vitamins B12 and D, which were above the cut-off level for deficiency in both intervention and control groups, and across baseline and endline surveys (see Table 4).

**Table 4:** Micronutrient status at baseline and endline comparing primary school children in intervention and control groups*.

Biochemical Indicators	Baseline mean (sd)		Endline mean (sd)	
	Intervention (191)	Control (177)	Intervention (189)	Control (162)
Hemoglobin (g/dL)	12.40 ± 0.91	12.48 ± 0.83	12.58 ± 1.08	12.14 ± 1.01
Plasma ferritin (ng/mL)	56.08 ± 30.96	60.57 ± 37.59	65.79 ± 30.21	64.55 ± 52.00
Folic acid/folate (ng/mL)	7.78 ± 3.08	5.42 ± 1.50	10.73 ± 4.30	4.86 ± 1.610
Vitamin B12 (pg/mL)	563.04 ± 223.39	519.93 ± 210.21	500.00 ± 196.86	456.37 ± 196.05
Plasma retinol (μmol/L)	24.20 ± 6.38	22.73 ± 6.43	26.41 ± 6.66	24.88 ± 6.15
Zinc (mg/L)	0.68 ± 0.12	0.69 ± 0.11	0.72 ± 0.10	0.71 ± 0.11
Iodine (μg/L)	108.62 ± 124.75	104.14 ± 110.05	135.11 ±147.26	148.10 ±137.09
Vitamin D (nmol/L)	53.27 ± 18.40	48.65 ± 18.21	46.70 ± 24.94	35.31 ± 15.68

* Values are mean± sd

To assess the biologically significant impact of the intervention, regression models were considered for different log-transformed micronutrients, with the variables selected (according to the conceptual framework) to represent immediate and underlying causes of malnutrition as covariates (Table 5). After adjusting for covariates, we found that students who received the intervention had significantly higher serum levels of all micronutrients except for ferritin, zinc and iodine. Levels of hemoglobin, folic acid, vitamin B12, retinol and vitamin D were1.01, 1.76, 1.1, 1.08 and 1.1 times higher respectively in the intervention group compared to the control group.

**Table 5:** Estimates of fitted regression models for selected micronutrients.

Covariates	log Hb	log Fer	log Fol	logB12	log Ret	log Zn	log I	logVitD
**Treatment**								
Control								
Intervention	0.014^c^	0.031	0.566^a^	0.100^a^	0.078^a^	-0.006	0.014	0.109^a^
**Round**								
Baseline								
Endline	-0.003	0.178^a^	0.108^a^	-0.107^a^	0.075^a^	0.050^a^	0.277^a^	-0.240^a^
Treat*Round								0.112^c^
**Sex of child**								
Male								
Female	-0.005	-0.060	0.000	0.055	0.026	-0.012	-0.09	-0.082^b^
**Age of child**	0.003	0.043^b^	-0.034^a^	-0.023	0.009	-0.008	-0.016	-0.021
**Mother’s**	-0.002	-0.005	0.006	0.003	0.013^a^	0.000	0.031^b^	0.005
**education**								
**H. pylori**	-0.004	-0.047	-0.067^b^	-0.07^c^	-0.04^c^	0.000	0.074	0.030
**C-reactive**	0.002	0.029^a^	0.001	0.006	-0.02^a^	0.000	-0.011	0.022^a^
**protein**								
**Food**								
Dal	0.005^a^							
DGLV	-0.002	-0.018^c^						
Milk							-0.045^b^	
YOFV				-0.018^a^				
Egg				0.016^b^		0.009^b^		-0.02
Meat								
Fish								0.015^b^
**Wealth Index**								-0.027^b^
Lowest								
Second	0.002	-0.079	0.013	0.071	0.003	-0.033^c^	-0.155	0.038
Middle	0.002	-0.247^a^	0.039	-0.018	0.000	-0.035^c^	-0.156	0.050
Fourth	0.003	-0.239^b^	0.031	-0.001	0.020	-0.013	-0.059	0.092
Highest	-0.001	-0.330^a^	-0.017	0.094	-0.003	0.000	-0.285^b^	0.114^c^
**Const.**	2.483^a^	3.776^a^	1.863^a^	6.366^a^	2.988^a^	-0.311^a^	4.556^a^	3.968^a^

DGLV = dark green leafy vegetables, YOFV = yellow orange fruits and vegetables, Hb = hemoglobin, Fer = Plasma Ferritin, Fol = Folic Acid, B12 = Vitamin B12, Ret = Plasma Retinol, Zn = Zinc, I = Iodine, VitD = Vitamin D;

^a^: p ≤ 0.01;

^b^: 0.01<p ≤ 0.05;

^c^: 0.05< p ≤ 0.1.

From baseline to endline, significant increases in levels of ferritin (1.19 times), folic acid (1.11 times), retinol (1.07 times), zinc (1.05 times), and iodine (1.32 times) were apparent. For Vitamins B12 and D, a significant decrease occurred across the study periods. The interaction between treatment and round was significant for vitamin D only. When comparing intervention and control groups, the decrease in average serum levels of vitamin D from baseline to endline was significantly less (1.12 times) for the intervention group compared to the control group.

No significant effect of gender was found except for vitamin D for which average serum levels were 1.08 times higher for boys compared to girls. Mothers' education was significantly associated with children’s serum retinol and iodine levels. An increase in one year of schooling among mothers was associated with a 1.3% and 3.1% increase in retinol and iodine, respectively.

H. pylori, an indicator of infection, had a significant negative association with folic acid, vitamin-B12, and retinol. The presence of H. pylori was associated with a decrease in the average level of these micronutrients by about 7% for folic acid and vitamin-B12, and about 4% for retinol-levels.

C-reactive protein was significantly associated with ferritin, retinol, and vitamin-D. A one unit increase of C-reactive protein corresponded to an average increase of 3% in ferritin and vitamin-D, and a 2% decrease in retinol.

We also considered separate log-binomial regression models [21] to estimate the treatment effect on different micronutrient deficiencies measured at endline, where corresponding baseline deficiency, students' gender, CRP and baseline H. pylori status are considered as covariates. Table 6 shows the observed prevalence of different micronutrient deficiencies at baseline and endline, and the adjusted relative risk and corresponding 95% confidence interval, which are obtained from the corresponding fitted log-binomial regression model using R package logbin [21]. The fits of log-binomial regression models show that the treatment has a significant effect (at 5% levels of significance) on reducing hemoglobin and vitamin D deficiencies. More specifically, the risk of developing anemia or vitamin D deficiency is about 32% smaller in intervention group students compared to control group students.

**Table 6:** The prevalence and adjusted relative risk ratios of different micronutrient deficiencies at baseline and endline comparing primary school children in intervention and control groups*.

	Baseline %		Endline %			
Outcome	Intervention	Control	Intervention	Control	Adjusted RR	95% CI
	(191)	(177)	(189)	(162)		
Anemia	28	20	26	31	0.670 ± 0.133	(0.408, 0.931)
Iodine deficiency	119	119	105	79	1.094 ± 0.108	(0.883, 1.305)
Vitamin D deficiency	42	60	80	101	0.677 ± 0.072	(0.536, 0.817)
Zinc deficiency	80	64	44	46	0.763 ± 0.134	(0.500, 1.025)

*Nutrient deficiencies with a small number of events are not presented here

### Qualitative results

The qualitative study considered the acceptability of the fortified biscuit intervention from the point of view of students and teachers. Despite efforts to minimize desirable answers, very few complaints were registered regarding the biscuit program, nor were any negative social or physical consequences associated with daily consumption.

#### School attendance and enrolment

According to teachers, since the initiation of the program school attendance has appeared to be more regular. As one teacher noted: *“At present*, *the attendance remains pretty high; previously*, *when the biscuits were not provided*, *the attendance was not like this*.” Moreover, during periodic stock-outs in several schools, a drop in attendance was reported. One headmaster explained:

*At the time when (biscuit) distribution was interrupted, some students became irregular; we then contacted with them and discussed about it; they started to attend school again when the distribution became regular*.

Some teachers also associated a rise in school enrolment and a decline in dropouts with the biscuit intervention. One teacher claimed that a rise in school admissions occurred compared to the previous year: *“There were 300 students in our school before*, *now it has increased up to 450/475*.*”* Perceived impacts on dropout were also noted:

*“We have noticed some of the students to drop out; but now they are coming to school again; their interest for attending school has increased much”*.

#### School performance

In addition to improvements in school attendance, some teachers noted greater concentration in the classroom:

*Their attention has increased; now they show interest and effort… Most of the families here are poor; they hardly can manage food. After the biscuit program started, they try to cover the lesson that we teach them, and complete the rest after they get back home*.

One teacher went so far as to attribute better student performance to the supplementation program, noting that:

*“When they perform well in the class and study well*, *they ought to (produce) good results; the number of failures has decreased at present*.*”*

Students perceived positive impacts on school performance, the biscuits reportedly helping them memorize study materials more easily, and doing better on their exams. One student explained the effect of the biscuit as “(giving) *energy to my brain; then it feels good to study*.*”*

#### Perceived health benefits

Many positive health outcomes were associated with biscuit consumption by teachers and students alike. Using body mapping techniques, students indicated feeling stronger in their hands and legs; and having a greater interest in running and playing. Students also reported that their stomach felt content, and their appetite had increased. One student noted that “*It feels like peace inside the belly*. *All the bones inside my chest feel well after having the biscuits*.*”*

Several teachers perceived that health complaints were less frequent: a teacher explained *“I have been working in this school for last 2 years; they (students) use to complain of fever*, *abdominal pain*, *diarrhea; now the complaints have decreased a lot*. *It is due to the biscuits*, *I guess*.*”*

#### Energy levels

Teachers also claimed that students were less hungry in the classroom and, according to guardian reports, when they returned home after school. Within the classroom they noted that children appeared livelier overall, and more engaged in learning:

*The children used to look dizzy in the class before*, *but it does not happen now; now they seem to be more active*.*”*

Similar reports came from students, with repeated remarks such as: *“*…*after the biscuits*, *I have peace inside my mind*.*”*

#### Socio-economic benefits

Another benefit mentioned by teachers is that after the program started, they noticed that parents were no longer providing pocket money to their children, and children were no longer buying tiffin (snacks) from shops near the schools—a likely financial relief for poor families. According to one teacher:

*All the students do not belong to families of similar socio economic status; about 85% of the students come from families who can’t afford a meal three times a day; this (biscuits) is like a support for those students*.

#### Acceptability

Qualitative findings indicated that students were consuming biscuits on a regular basis. According to students, the biscuits were very tasty, a number sharing a popular practice of pulverizing the biscuits into powder, “(*pouring) the powder to our water bottles and saying that we are drinking Horlicks*.*”* Teachers confirmed that students consume the biscuits willingly and enthusiastically. As one teacher explained:

*When the supply was interrupted several days ago, some of the students thought that we were doing this purposively; we had to show them the empty box…*.

Another teacher remarked:

*When we are a few minutes late in distributing the biscuits among the students, some of them become very impatient and ask us, Madam, wouldn’t you give us the biscuits today*?

According to one teacher, it is common for students to ask for the biscuits even when they are sick or have to leave the school early before biscuits are distributed.

## Discussion

While rates of child under-nutrition are improving in Bangladesh, levels of stunting remain very high with a prevalence of 35% among children under five [2]. Nutritional deficits accrued during early childhood can persist into the school-aged years, with implications for subsequent development including school performance [5]. Baseline micronutrient status in the primary school children participating in our study suggest worrisome levels of deficiency which are in line with recent findings from the National Micronutrient Survey (NMS) [4]: rates of anemia were slightly lower (12.5% vs. 19.1%); Vitamin A deficiency was slightly higher (36% vs. 20.9% in MNS), while levels of iron deficiency anemia measured by serum levels of ferritin were similar (5.5% vs. 3.9%). Socioeconomic variation may account for small differences as the pilot supplementation program was undertaken in areas of Bangladesh with greater vulnerability to food insecurity [2].

It is well established that investments in nutrition and stimulation programs during the first two years of life are key to optimizing child growth and development [1]. While school feeding programs cannot reverse the damage of early nutritional deficits, evidence from systematic reviews identify measurable impacts of feeding and fortification approaches on micronutrient deficiencies, school attendance, and the growth of school-age children [6, 24]. This study is among the first in Bangladesh to comprehensively assess a school-based fortification program in terms of its acceptability and impact on the micronutrient status of children aged 6–11 years of age. Daily consumption of fortified biscuits by primary school children was found to have a significant positive impact on mean levels of hemoglobin, folic acid, vitamin B12, retinol and vitamin D, and helped reduce anemia and vitamin D deficiency.

Similar positive results have been noted in fortification studies in other parts of Asia and Africa. In Vietnam, for example, a recent evaluation study compared the effectiveness of daily fortified biscuits (of similar composition to those in our study) and weekly iron supplement tablets delivered to school-going children aged 6–9 years of age [9]. Six months later, a significant reduction in anemia and improved iron status was recorded among students receiving fortified biscuits, whereas the group receiving iron tablets had improved iron status only [9]. A randomized placebo-controlled trial among children aged 6–11 years in South Africa also showed that fortified biscuits reduced the prevalence of anemia, as well as vitamin A, and iodine deficiencies [7]. This study involved the provision of a Vitamin C fortified drink on daily basis along with iron-fortified biscuits to enhance the absorption of iron. Followed up at 12 months, significant reductions in deficiency were noted. In Kenya, a significant improvement in iron status and anemia was found in children aged 3–8 years consuming iron-fortified maize porridge over a 5 month period compared those in the control group [25].

Similar to the studies above, our research findings show improvements in levels of anemia in the intervention group. While the associations between iron, cognition and learning achievement are well known [26], a recent review suggests that some long term negative consequences of early-life iron deficiency may be irreversible, and even those above the cut-off for anemia, can suffer adverse consequences [27]. These findings provide compelling justification for iron fortification to minimize the cognitive and behavioral consequences of deficiency among young children [27, 28]. Also of interest were sex differences in Vitamin-D, with higher serum levels apparent among primary school-going boys in both intervention and control groups. Consistent with another study in Saudi Arabia, this may be culturally mediated, and related to greater sun exposure among boys than girls, resulting in more vitamin D being produced [29].

Negative associations between the presence of H. pylori and levels of folic acid and vitamin B12 were also seen in our study which is consistent with evidence that the absorption of iron and vitamin B12 is impaired by H. pylori infection in humans [30, 31], and accordingly, with studies linking H. pylori eradication with improved serum levels of vitamin B12 and vitamin A [32]. H pylori eradication also reduces levels of iron deficiency anemia, and should be considered an important complement to micronutrient fortification interventions for young children. The effect of inflammation on micronutrient status, such as ferritin and retinol, is also well recognized in the literature. While we controlled for C-reactive Protein in our analyses, a detailed examination of its influence lies beyond the remit of this paper.

Qualitative findings provided strong evidence of the acceptability of the fortified biscuit intervention by both teachers and students. Teachers perceived students to be more attentive in class, less tired, and some attributed better school performance to the intervention. These findings are consistent with other school-based fortification evaluations in Bangladesh [10]. In our study, children’s impressions of the fortified biscuits were also explored, and revealed similar perceived improvements in concentration and energy levels. The timing of biscuit consumption appeared to be an important factor underlying its perceived impact on attentiveness in the classroom, providing energy and nutrients at a critical point in the school day. However, the extent to which changes in perceived concentration, memory and motivation are due to the energy content of the biscuit, or to micronutrient fortification and its cognitive impacts, is impossible to assess.

## Study limitations

Several limitations should be mentioned that may have impacted the sensitivity of the study to changes in micronutrient levels, and the attribution of these changes to the fortification program. First is the confounding influence of seasonality in dietary intake from baseline to endline. The availability of seasonal fruits and vegetables may have a large effect on the micronutrient status of the school going children irrespective of fortification. A second limitation concerns the timing of baseline biological measures. Biscuits were initially received in early August for several weeks, then distribution was suspended during a one-month Holiday period. The survey team started their work immediately following the holiday, and biological samples were collected two months later. While it is likely that serum levels in children would require several months to respond to the effects of micronutrient fortification, this delay may have resulted in an underestimation of the effect of intervention. Comparison with the control group, however, suggests that these effects were minimal. Third was the absence of a placebo group, making it difficult to assess the extent to which perceived improvements in attentiveness and concentration was due to the energy content of the biscuit, or to the micro-nutrients it contains. Finally, it is important to note that use of biscuits for fortification school-based food programs may not be the most ideal approach to provide micronutrients to disadvantaged populations. FAO recommends that ingredients for school feeding programs be purchased from local farmers, and that locally preferred food be provided to learners instead of processed foods such as biscuits [33]. Further, the biscuits contained 12gms of free sugar. The WHO recommendation states that free sugar should not exceed 10% of the total calorie intake. Given that we do not know the calorie intake of the population, it unclear whether 12 gms of sugar/ 100gms of biscuits exceeds recommended levels [34].

## Conclusions

Contributing to the growing literature on benefits of school-based fortification programs, our study strongly supports their acceptability and micronutrient impact among primary school children in Bangladesh. Given that primary school attendance is about 92% (2010) [35], school-based fortification approaches offer an efficient strategy for addressing micronutrient malnutrition among young children. Moreover, school-based delivery in the form of easily distributed fortified biscuits allowed substantial coverage of primary school aged children, and strong compliance due to daily contact. Given the overwhelming popularity of the fortified biscuits, and their proven effectiveness in terms of improving micronutrient status, school-based interventions of this nature, may have considerable advantages over community-based or health facility modes of delivery. Indeed it can be argued that costs associated with biscuit production and distribution are more than offset by the short and long term benefits of the program—providing energy and nutrients that help optimize children’s learning in the classroom, and their later capabilities as parents and contributors to the national economy. While the advantages of food-based approaches are acknowledged, the popularity and effectiveness of this mode of delivery are clear. The extent to which micronutrient fortified biscuits yield measurable cognitive impacts will be important to assess to make an even stronger case for scaled-up investments in school- based feeding programs in Bangladesh and elsewhere.

## Supporting information

S1 Appendix Micronutrients and their influence. (DOCX)

S2 Appendix Biochemical measures. (DOCX)

S3 Appendix Data set. (CSV)

## Abbreviations

BMI: Body Mass Index
FGD: focus group discussions
GEE: Generalized Estimating Equation
Hb: Haemaglobin
icddr,b: International Centre for Diarrhoeal Disease Research, Bangladesh
