# Supplementary material for: Impact of fortified biscuits on micronutrient deficiencies among primary school children in Bangladesh
Source: PLoS One. 2017 Apr 5;12(4):e0174673. doi: 10.1371/journal.pone.0174673 (PMC5381786; doi:10.1371/journal.pone.0174673)
Supplement: S1 Appendix — (DOCX) [file pone.0174673.s001.docx]

**S1 Appendix. Micronutrients and their influence**

**Iron** transports oxygen as a component of hemoglobin red blood cells, and is required for certain reactions involving energy formation. Iron deficiency and iron deficiency anemia are manifested by weakness, fatigue and reduced attention span and resistance to infection. Supplementation during childhood has been shown to improve growth, immune function, motor and cognitive development of the growing child.

**Zinc** is important for growth and development of school aged children, because of its role in immune function and the prevention of diseases. Zinc deficiency is accompanied by growth failure, delayed sexual maturation, loss of appetite, and slow wound healing. Zinc supplementation is now part of the standard treatment for diarrhea among children.

**Folate/Folic acid** is an integral part of DNA, RNA and protein synthesis. It is required for the normal formation of red blood and other cells. School aged children and adolescents have increased requirements for folate in their body. Deficiency is linked to megaloblastic cells and anemia, diarrhea, weakness, irritability and paranoid behavior.

**Vitamin E** acts as an antioxidant, preventing damage to cell membranes in blood cells, lungs and other tissues. Reduces oxidation of LDL cholesterol. Deficiency symptoms are muscle loose, nerve damage, anemia, and weakness.

**Vitamin C** is required for collagen, neurotransmitter and steroid hormone synthesis, and acts as an antioxidant. Deficiency is manifested in easy bleeding and bruising does, slow recovery from infections and poor wound healing.

**Iodine** helps to regulate energy production and growth. Deficiency of Iodine in school aged children lead to mental retardation, hearing loss and growth failure.

**B12** is involved in the synthesis of DNA, RNA and myelin and needed for normal red blood cell development; prevents neurological disorders, anemia and fatigue.

**Vitamin D** is required for calcium and phosphorus metabolism in the intestine and bone and also needed for teeth formation, nerve and muscle activity. Deficiency of Vitamin D in school aged children leads to weak, deformed bones.
