# Supplementary material for: Impact of fortified biscuits on micronutrient deficiencies among primary school children in Bangladesh
Source: PLoS One. 2017 Apr 5;12(4):e0174673. doi: 10.1371/journal.pone.0174673 (PMC5381786; doi:10.1371/journal.pone.0174673)
Supplement: S2 Appendix — (DOCX) [file pone.0174673.s002.docx]

**S2 Appendix: Biochemical Measures**

| *Assessments of Hemoglobin*  To measure hemoglobin levels in participating children, approximately 2 ml of blood was obtained by venipuncture using aseptic technique. 0.5 ml of collected blood was placed in an Eppendorf tube with EDTA. The blood was then smoothly shaken to mix with the anticoagulant homogenously for Hb measurement. Within 8-12 hours of collection, the samples were transported to the Nutritional Biochemistry Laboratory of icddr,b for analysis in temperatures maintained between 4 and 8° C. Blood samples were stored at -20° C until analyzed. Hb was measured spectrophotometrically (SIGMA, St Louis, MO kit) after conversion of Hb to cyanmethemoglobin (Drabkin’s solution method).  *Other biochemical tests* were performed on a separate sample of blood (1.5 mL). These include:  *Assessments of C-reactive Protein (CRP)*  CRP was measured by Immunoturbidimetric methods using a hitachi-902 autonanalyzer (Boehringer Mannheim, Germany) and Tina-quant CRP kit (Roche Diagnostics, GmbH, D-68298 Mannheim). Tina-Quant CRP Assay is an immunoturbidimetric test for the quantitative determination of CRP in human serum and plasma. During the reaction, anti- CRP antibodies coupled with latex micro-particles reacted with CRP in the sample to form an antigen-antibody complex. Following agglutination, the precipitate was measured turbidimetrically. CRP concentration was proportional to the amount of turbidity formed. Commercially available Precinorm and Precipath Protein (Roche Diagnostics, GmbH, D-68298 Mannheim) was used for checking quality control. We also used pool serum as internal quality control. To ensure quality control, we participate in CDC’s vital External Quality Assessment (EQR) programme for serum CRP.  *Assessments of Serum Ferritin*  Ferritin was measured by Immunoturbidimetric methods using hitachi-902 autonanalyzer (Boehringer Mannheim, Germany) and the Tina-quant ferritin kit (Roche Diagnostics, GmbH, D-68298 Mannheim). The Tina-Quant Ferritin Assay is an immunoturbidimetric test for the quantitative determination of ferritin in human serum and plasma. During the reaction, anti-ferritin antibodies coupled with latex microparticles react with ferritin in the sample to form an antigen-antibody complex. Following agglutination, the precipitate was measured turbidimetrically. Ferritin concentration was proportional to the amount of turbidity formed. Commercially available Precinorm and Precipath Protein (Roche Diagnostics, GmbH, D-68298 Mannheim) were used for checking quality control. We also used pool serum as internal quality control. To assure quality control, we participate in CDC’s EQA programme for serum ferritin.  *Assessments of Retinol*  Serum/plasma was deproteinized and extracted into hexane. The hexane layer was evaporated and re-dissolved in the mobile phase and injected into HPLC column. Retinol was separated by reverse phase HPLC using C18 column and detected at 325nm. A pooled human serum sample was calibrated against standard reference material (fat-soluble vitamins, carotenoids and cholesterol in human serum, 968c; National Institute of Standards and Technology, Gaithersburg, MD, USA). Three aliquots of the serum pool were analyzed with each set of samples, and the retinol concentration was calculated based on the known concentration of retinol in the serum pool. For quality control, we participate in different EQA programmes (NIST, CDC) for serum retinol annually.  *Assessments of Folic Acid*  Serum sample was incubated with the folate pre-treatment reagents to release bound folate from endogenous folate binding proteins. Then, the pretreated sample was incubated with the ruthenium labelled folate binding protein and a folate complex is formed, the amount of which is dependent upon the analytic concentration in the sample. Next, streptavidin-coated microparticles and folate labelled with biotin were added and the unbound sites of the ruthenium labelled folate binding protein became occupied, with formation of a ruthenium labelled folate binding protein-folate biotin complex. The entire complex was bound to the solid phase via interaction of biotin and streptavidin. The reaction mixture was then aspirated into the measuring cell where the microparticles were magnetically captured onto the surface of the electrode. Unbound substances were washed away and application of a voltage to the electrode induced chemiluminescentemission which was measured by a photomultiplier. Results were determined via a calibration curve. For quality control, we used Pool serum (prepared in the Nutritional Biochemistry Laboratory), ElecsysPreciControl Anemia 1, 2 and 3 (Roche Diagnostics). We established mean and SD by analyzing each QC material in 10 replicates within same day and establish quality control chart by using mean ± 2SD. We also participate in CDC vital EQA programme for serum folate.  *Assessments of Vitamin B12*  Serum/Plasma was incubated with vitamin B12 pre-treatment reagent to release bound vitamin B12. By incubating the pre-treated sample with the ruthenium labelled intrinsic factor, a vitamin B12-binding protein complex was formed, the amount of which was dependent upon the analytic concentration in the sample. After addition of streptavidin-coated microparticles and vitamin B12 labelled with biotin, the still-vacant sites of the ruthenium labelled intrinsic factor became occupied, with formation of a ruthenium labelled intrinsic factor-vitamin B12 biotin complex. The entire complex became bound to the solid phase via interaction of biotin and streptavidin.  The reaction mixture was aspirated into the measuring cell where the microparticles were magnetically captured onto the surface of the electrode. Unbound substances were then removed with ProCell. Application of a voltage to the electrode then induced chemiluminescent emission which is measured by a photomultiplier. Results are determined via a calibration curve which was instrument-specifically generated by 2-point calibration and a master curve provided via the reagent barcode. For quality control, we used ElecsysPreciControl Anemia 1, 2 and 3. We also used pool serum as internal quality control, and for external assessment we participate in CDC’s EQA programme for serum vitamin B12  *Assessments of Zinc*  Serum was diluted and aspirated directly into the AAS flame. We calculated the results from the standard curve. Bi-level control serum (normal and high range) (UTAK Laboratories Inc, USA) were used to check the accuracy for serum zinc. We also used pooled serum in every lot to check for both accuracy and precision.  *Assessments of* 25-*Hydroxy Vitamin*  D25-Hydroxy Vitamin D was measured by Enzymeimmunoassay using IDS 25-Hydroxy Vitamin D EIA Kit. (Source: IDS Ltd, 10 Didcot Way, Boldon Business Park, Boldon, UK, www.idsplc.com). Calibrators, controls and samples were diluted with biotin labelled 25-Hydroxy Vitamin D. The diluted samples were incubated in microplate wells which were coated with a highly specific sheep 25-OH D antibody for 2 hours at room temperature. The plates were washed and enzyme (horseradish peroxidase) labelled avidin, was added and binded selectively to complex biotin and following a further wash, step colour was developed using a chromogenic substrate (TMB). The absorbance of the stopped reaction mixtures were read in a microtitre plate recorder, colour intensity developed being inversely proportional to the concentration of 25-OH D. A two level control set (IDS, UK) were used as quality control.  *Assessments of Urinary Iodine*  Using a specially designed sealing cassette to prevent loss of vapour and cross-contamination among wells, ammonium persulfate digestion was performed in a microplate in an oven at 110 °C for 60 min. After the digestion mixture was transferred to a transparent microplate and the Sandell–Kolthoff reaction was performed at 25°C for 30 minutes, urinary iodine was measured by a microplate reader at 405 nm. For quality control, we used certified reference materials (CDC). We also participate in Ensuring the Quality of Iodine Procedures (EQUIP) which is a standardization programme that addresses laboratory quality-assurance issues related to testing for iodine deficiency. |
| --- |
